# Serial monitoring of LV function following chemotherapy: assessment using advanced echocardiography and cardiovascular magnetic resonance

**DOI:** 10.1186/1532-429X-16-S1-P139

**Published:** 2014-01-16

**Authors:** Suchi Grover, Rebecca Perry, Darryl Leong, Majo Joseph, Bogda Koczwara, Joseph Selvanayagam

**Affiliations:** 1 Flinders University, Adelaide, South Australia, Australia; 2 Cardiology, Flinders Medical Centre, Adelaide, South Australia, Australia; 3 Flinders Cardiac Cardiovascular Magnetic Resonance, Flinders Medical Centre, Adelaide, South Australia, Australia; 4 Medical Oncology, Flinders Centre for Innovation in Cancer, Adelaide, South Australia, Australia

## Background

Patients receiving anthracyclines (A) and herceptin (H) for treatment of breast cancer require serial monitoring of LV function. Although CMR is considered the gold standard for assessment of volumes and function, most tertiary institutions rely on echocardiography or gated heart pool scans. 2D transthoracic echocardiographic (TTE) image quality relies on appropriate acoustic windows, and accuracy and reproducibility may be particularly limited in this population due to mastectomy and scar. We sought to assess the utility of TTE (LVEF and GLS) compared to CMR in the diagnosis of Stage B heart failure (defined as evidence of structural disease but without signs and symptoms of heart failure [1]) in breast cancer patients receiving cytotoxic chemotherapy.

## Methods

In total, 44 patients (30 receiving A and 14 receiving T) underwent cardiac magnetic resonance (CMR) for LV function and advanced 2D echocardiography for LV function and global longitudinal strain (GLS) at 3 time-points (baseline, 3 months and 12 months). We defined stage B heart failure as LVEF < 10% from baseline, and/or LVEF below 55% and/or GLS < -19.7[2].

## Results

CMR cine assessment of LV function was performed in all 44 patients (100%). Thirty percent (13/44) could not have accurate echocardiographic EF assessment performed at 1 or more time points. Early LV dysfunction was detected by CMR following commencement of chemotherapy (table). There was poor correlation between CMR LVEF and echocardiography EF (graph) with wide limits of agreement (-23.526 to 13.015%). At 12 months, 8 patients (18%) demonstrated stage B heart failure, however none were below the lower limits of normal (>55%). None of these showed a significant change in TTE EF, however 2 of these patients had GLS < 19.7 at 12 months.

## Conclusions

A significant number (approx. 1/3) of breast cancer patients are unable to have adequate LV function assessment by echocardiography. Subtle LV dysfunction may be a precursor to overt cardiomyopathy and is currently not adequately diagnosed by echocardiography even with inclusion of 2D global longitudinal strain. Serial monitoring of LV function using a non-radiation imaging technique is best performed by CMR.

## Funding

None.

**Table 1:** 

	LVEF (CMR) (%)	LVEF (Echo) (%)	GLS
Baseline	71.9 ± 6.3	63.8 ± 5.7	-21.4 ± 2.6

3 months	66.7 ± 6.7 **	63.5 ± 7.2	-19.5 ± 2.0 **

12 months	65.9 ± 7.0 **	62.1 ± .9	-20.1 ± 2.6

**Figure 1:**
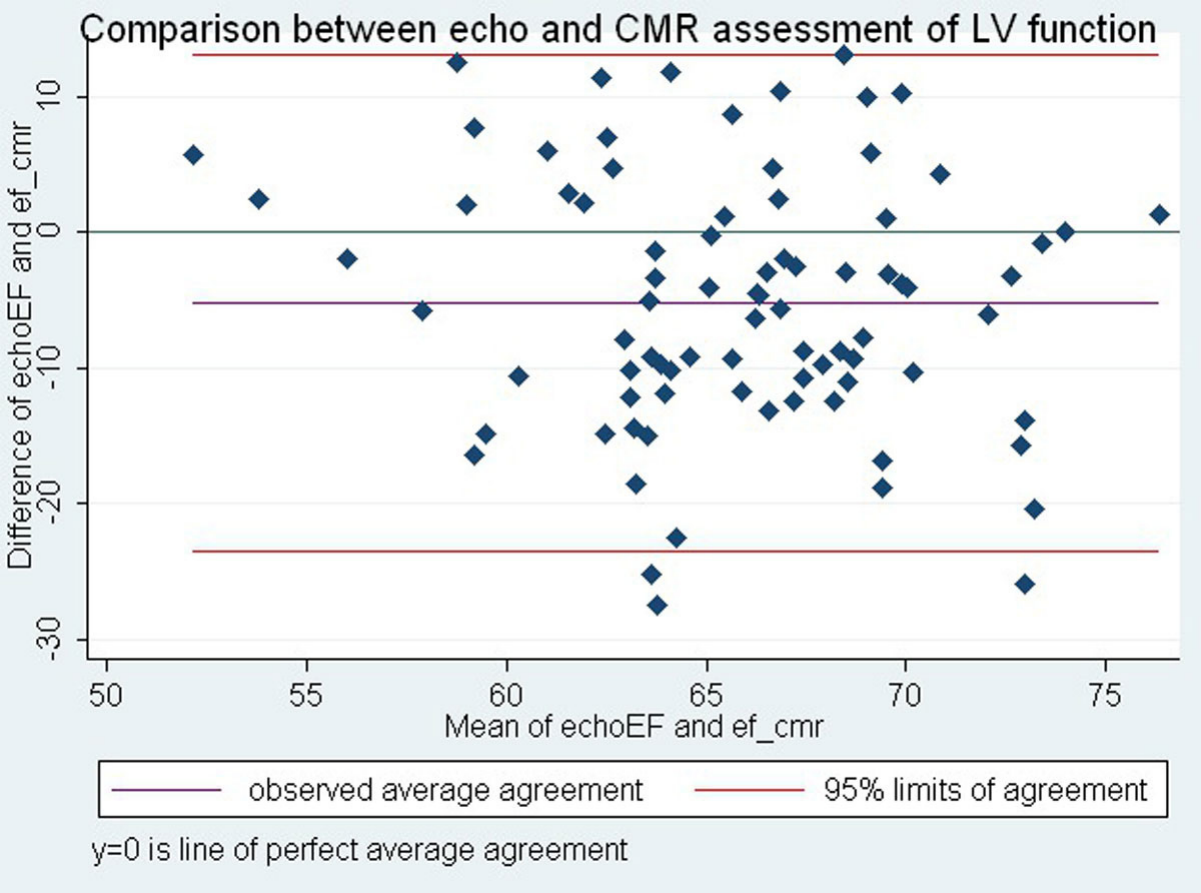


## References

[B1] Hunt SA Abraham WT Chin MH Feldman AM Francis GS Ganiats TG 2009 focused update incorporated into the ACC/AHA 2005 Guidelines for the Diagnosis and Management of Heart Failure in Adults: a report of the American College of Cardiology Foundation/American Heart Association Task Force on Practice Guidelines: developed in collaboration with the International Society for Heart and Lung Transplantation Circulation 2009 119 14 e391 479 19324966 10.1161/CIRCULATIONAHA.109.192065

[B2] Yingchoncharoen T Agarwal S Popovic ZB Marwick TH Normal ranges of left ventricular strain: a meta-analysis J Am Soc Echocardiogr 2013 26 2 185 91 10.1016/j.echo.2012.10.008 23218891

